# Assessing the prevalence of anosmia and taste dysfunction in COVID-19 patients in Saudi Arabia: a systematic review and meta-analysis

**DOI:** 10.3389/fpubh.2026.1815114

**Published:** 2026-07-21

**Authors:** Turki Aldrees

**Affiliations:** Department of Otolaryngology–Head and Neck Surgery, College of Medicine, Prince Sattam Bin Abdulaziz University, Alkharj, Saudi Arabia

**Keywords:** anosmia, COVID-19, gustatory dysfunction, meta-analysis, olfactory dysfunction, prevalence, SARS-CoV-2, Saudi Arabia

## Abstract

**Objectives:**

Patients with coronavirus disease-2019 (COVID-19) frequently report olfactory and gustatory dysfunctions, including anosmia and ageusia. Multiple studies have assessed the prevalence of these symptoms in COVID-19 patients in Saudi Arabia. This systematic review and meta-analysis aimed to estimate the pooled prevalence of anosmia and taste dysfunction in confirmed COVID-19 patients in Saudi Arabia across inpatient, outpatient, and community settings, from March 2020 to March 2023.

**Methods:**

A systematic review and meta-analysis was conducted in accordance with the Preferred Reporting Items for Systematic Reviews and Meta-Analyses (PRISMA 2020) guidelines. No review protocol was prospectively registered prior to this review. A comprehensive search was conducted across PubMed, ScienceDirect, CINAHL, MEDLINE, and Google Scholar (supplementary) from 1 March 2020 to 1 March 2023. Data were pooled using a random-effects model. The methodological quality of included studies was evaluated using the Joanna Briggs Institute (JBI) critical appraisal tools for prevalence studies.

**Results:**

Of 230 records identified across all databases, 156 remained after deduplication; 24 met the eligibility criteria, encompassing 26,068 COVID-19 patients (58.2% female). The pooled prevalence of anosmia was 44% (95% CI: 37–52%) and of taste dysfunction was 46% (95% CI: 39–54%). Subgroup analyses comparing validated versus non-validated assessment instruments revealed no statistically significant difference in prevalence estimates.

**Conclusion:**

Anosmia and taste loss are common manifestations of COVID-19 in Saudi Arabia. The findings should be interpreted with caution given the methodological heterogeneity of included studies, reliance on self-reported measures in the majority of studies, and the absence of a prospectively registered protocol. Future studies using objective validated instruments are warranted.

## 1 Introduction

The coronavirus disease-2019 (COVID-19) outbreak posed unprecedented challenges for healthcare systems worldwide. Early symptoms of COVID-19, primarily reported in hospitalized patients, include cough, shortness of breath, fever, and lower respiratory tract infections (1). Official reports have also described nasal congestion, sore throat, myalgia, rhinorrhoea, and fatigue. As evidence accumulated, smell and/or taste dysfunction emerged as frequently reported symptoms in patients with COVID-19, ranging from a reduced sense of smell or taste to complete loss of olfactory and gustatory function (2). Based on this evolving evidence, the World Health Organization included smell and taste loss in its official list of COVID-19 symptoms.

The loss of olfactory and gustatory functions significantly affects quality of life, resulting in malnutrition, food poisoning, weight loss, and depression (3). Healthcare providers have implemented olfactory training programs to facilitate recovery of olfactory function (4). The American Academy of Otolaryngology included hyposmia, anosmia, and dysgeusia among the screening symptoms for COVID-19, as loss of smell can be an early indicator of infection (5).

Studies conducted in Saudi Arabia have identified anosmia as a major symptom of COVID-19; however, no prior systematic review has robustly synthesized this evidence at the national level. The present review addresses this gap by answering the following structured question: Among confirmed COVID-19 patients in Saudi Arabia across inpatient, outpatient, and community/survey settings what is the prevalence of anosmia and taste dysfunction from March 2020 to March 2023? To the best of our knowledge, this is the first systematic review and meta-analysis to estimate the pooled prevalence of anosmia and taste dysfunction in COVID-19 patients in Saudi Arabia.

## 2 Methods

This systematic review and meta-analysis was conducted and reported in accordance with the Preferred Reporting Items for Systematic Reviews and Meta-Analyses (PRISMA 2020) guidelines. No review protocol was prospectively registered prior to this review; this limitation is discussed in section 4.1.

### 2.1 Eligibility criteria

Studies were eligible for inclusion if they met the following criteria: Studies were eligible if they reported the prevalence of anosmia and/or taste dysfunction (gustatory dysfunction) in patients with confirmed COVID-19 (by RT-PCR or clinical diagnosis per WHO criteria) in Saudi Arabia, published between 1 March 2020 and 1 March 2023. Included studies spanned three care settings: inpatient (hospitalized patients), outpatient (clinic-based), and community/survey-based cohorts.

### 2.2 Inclusion criteria

- Studies published between 1 March 2020 and 1 March 2023.
- Studies reporting the prevalence of anosmia and/or gustatory dysfunction in COVID-19 patients.
- Empirical (primary) studies only cross-sectional, cohort, and retrospective study designs. Review articles were excluded.
- Studies conducted in Saudi Arabia. Studies from any care setting (inpatient, outpatient, or community/survey) were eligible.

### 2.3 Exclusion criteria

- All types of review articles.
- Studies conducted outside Saudi Arabia.
- Studies assessing anosmia or gustatory dysfunction in conditions other than COVID-19.

### 2.4 Search strategy

PubMed, ScienceDirect, CINAHL, MEDLINE, and Google Scholar were searched for studies published between 1 March 2020 and 1 March 2023. Google Scholar was searched as a supplementary source; all records retrieved were screened using the same eligibility criteria as records from formal databases. Multiple keywords were combined using Boolean operators (AND, OR, NOT). Full search strings, search dates, language restrictions (English only), and per-database record counts are provided in Supplementary Table S1. The full search strings used for each database are presented in Supplementary Table S1. Key search terms included: COVID-19, SARS-CoV-2, anosmia, loss of smell, olfactory dysfunction, hyposmia, parosmia, loss of taste, ageusia, hypogeusia, gustatory dysfunction, prevalence, incidence, and Saudi Arabia, combined in various Boolean strings.

Supplementary search methods: Manual reference checking of all included articles and relevant systematic reviews was performed to identify any additional eligible studies. No gray literature databases (e.g., OpenGrey, PROSPERO) or clinical trial registers were searched, and no contact with study authors was made.

### 2.5 Study selection

**Figure 1:**
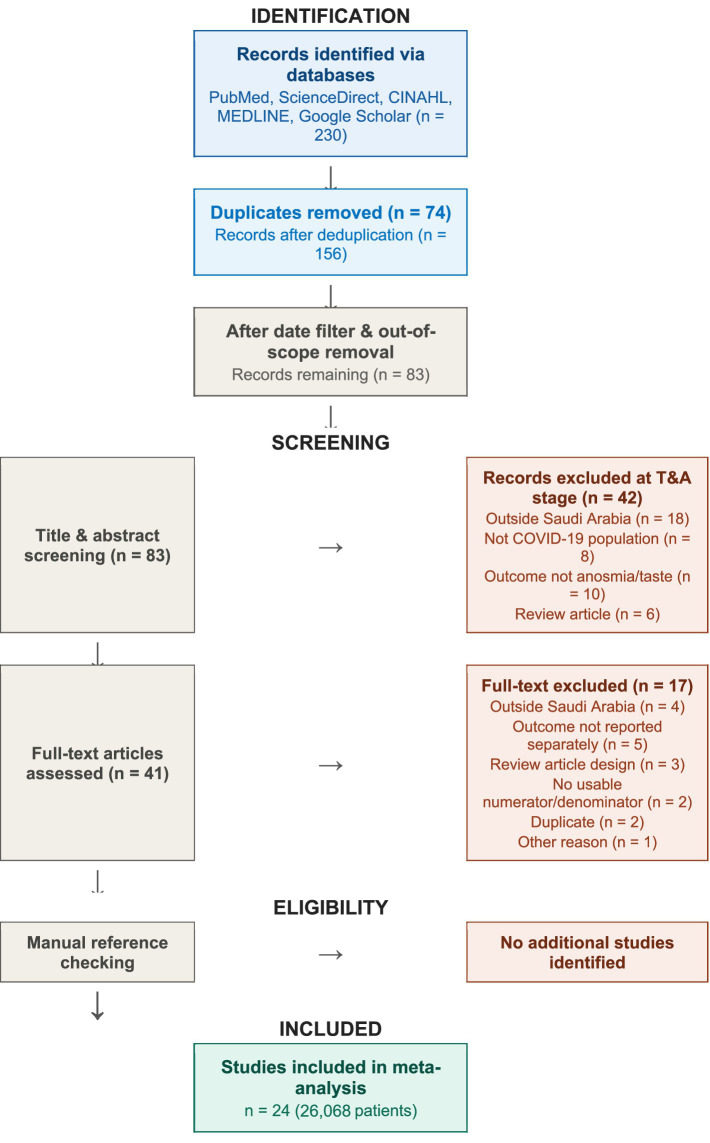
PRISMA 2020 flow diagram. Google Scholar was searched as a supplementary source using the same eligibility criteria as formal databases.

Study selection was performed primarily by T. A., with verification checks conducted by S. A. on a random sample of approximately 30% of records at each stage to identify errors or omissions. At Stage 1 (title and abstract screening), records were assessed for three criteria: (1) COVID-19 patient population, (2) Saudi Arabia study setting, and (3) reporting of anosmia or taste dysfunction as an outcome. Records clearly not meeting these criteria were excluded. At Stage 2 (full-text review), all pre-defined inclusion and exclusion criteria were applied in full to the remaining records. Disagreements at either stage were resolved through discussion and consensus. The PRISMA 2020 flow diagram (Figure 1) presents the number of records at each stage, including specific reasons for full-text exclusion.

### 2.6 Data extraction

Data extraction was performed by T. A. using a pre-specified Excel spreadsheet. S. A. independently verified extracted data for a random sample of approximately 30% of included studies to check for errors or omissions. Discrepancies identified during verification were resolved by discussion between the two reviewers. The following variables were extracted: author name, publication year, study design, total participants, sex, age, numerator and denominator for anosmia prevalence, and numerator and denominator for taste dysfunction prevalence (if reported in the studies), recovery status, hospitalization history, duration of symptoms, smoking status, instrument used to measure olfactory/gustatory function, and comorbidities.

### 2.7 Outcome definitions and timepoint prioritization

The primary outcomes were: (1) prevalence of anosmia (complete or partial loss of smell) and (2) prevalence of taste dysfunction (ageusia or hypogeusia) among COVID-19 patients in Saudi Arabia.

Both validated instruments [e.g., Quick Smell Identification Test (Q-SIT), University of Pennsylvania Smell Identification Test (UPSIT), Sniffin’ Sticks, Pocket Smell Test, sQODNS, AAO-HNS criteria] and non-validated instruments (self-administered questionnaires, structured telephone interviews, medical record-based coding) were accepted, reflecting the diversity of assessment methods in the included literature. Studies using validated and non-validated instruments were treated separately in a pre-planned subgroup analysis (see section 2.8).

Time point prioritization: When a study reported prevalence at multiple timepoints, the measurement closest to symptom onset or the acute phase of infection was prioritized. Where only recovery-phase or long-term data were available, these were used and noted accordingly in Table 1.

**Table 1:** Major characteristics of included studies.

Author	Total participants	Female (n)	Anosmia prevalence n/N (%)	Taste disturbance prevalence n/N (%)	Recovery	Age_(mean)	Hospitalization	Duration of anosmia/taste loss	Smoking status	Tool for olfaction detection	Comorbidities	City
Bin Abdulrahman et al. (8)	223	67	74/223* (33.20%)	70/223* (31.40%)	100%	32.5	NR	NR	27.40%	Online survey	NR	Riyadh
Albaharna et al. (9)	270	72	44/270* (16.30%)	86/270* (31.90%)	NR	43	88.00%	4 days, 5 days	8.50%	Validated (Q-SIT)	NR	Qatif
Alfallaj et al. (10)	1,255	484	853/1255* (68.00%)	715/1255* (57.00%)	Complete: 86% Partial: 13% No recover: 1%	38.26	15.53%	18.42 days	15.20	Survey	Heart condition: 4.8% Diabetes: 7.3% Combined heart and diabetes: 7.9% Chronic lung disease: 8.1% Autoimmune diseases: 1.1% Chronic renal 9 failure: 1.0%	Riyadh
Algahtani et al. (11)	808	445	582/808* (72.00%)	519/808* (64.20%)	NR	NR	4.60%	15–21 days	18.8%	Self-administered survey	Intestinal disease: 23.8% Diabetes: 8.3% Asthma: 7.8% Hypertension: 6.4%	Riyadh, Makkah, Al-Jouf and Al-Madinah
AlHazmi et al. (12)	174	130	20/174* (11.5%)	74/174* (42.5%)	NR	NR	NR	64.3 ± 110.3 days	NR	Self-administered survey	IBS: 1.1% Allergy: 5.7% Autoimmune disease: 1.7% Depression: 4.6% Heart problems: 1.1% Asthma: 6.3% GR: 4.6% Respiratory insufficiency: 1.1% Renal failure: 0.6% Hypothyroidism: 5.7% High blood pressure: 3.4% Diabetes: 5.7%	Al-Qassim region
Almandili (13)	544	290	234/544* (43.00%)	252/544* (46.30%)	91.7%	NR	NR	NR	NR	Questionnaire	Diabetes: 17.4% Hypertension: 25% Cardiovascular disease: 13.4% Cancer: 5.2% Renal diseases: 18.6% Respiratory disease: 26.2% Others: 18.6%	Taif
Alqahtani et al. (14)	81	39	51/81* (63.00%)	NR	NR	36.1	17.30%	NR	NR	Yes/no question (non-validated)	NR	Riyadh
Sara and Najlaa (15)	380	152	215/380* (56.60%)	215/380* (56.60%)	100%	38	NR	10 days	55.7%	Validated (AAO-HNS)	Diabetes: 40% Hypertension: 58.3% Head trauma: 42.9% Sinusitis: 58.5% Asthma: 50% Cardiac disease: 54.5%	Jeddah
AlRadini et al. (16)	1,000	395	550/1000* (55.00%)	490/1000* (49.00%)	NR	36.9	6.40%	28·3 + 34·7	20.1%	Questionnaire	Diabetes: 9.7% Hypertension: 7.9% Asthma: 5.7% Hyperparathyroidism: 2.3% Allergic disorders: 1.8%	Riyadh
Alroqi Ahmad et al. (6)	1,005	639	555/1,005* (55.22%)	NR	NR	NR	1.00%	1 week	NR	Self-administered survey	NR	Riyadh
Alrusayyis et al. (29)	257	101	175/257* (68.00%)	175/257* (68.09%)	100%	34.58	NR	4.81 days	19.07%	Validated survey	Diabetes: 9% Obesity: 29.1%	Dammam
AlShakhs et al. (17)	274	150	179/274* (65.3%)	177/274* (64.6%)	NR	NR	NR	NR		Validated (sQODNS)	NR	AlAhsa
Alshami et al. (27)	128	69	28/59* (47.5%)	61/128* (47.50%)	NR	39.6	NR	17 days	15.5%	Clinical case report form (non-validated)	Chronic lung disease: 2.3% Diabetes Mellitus: 13.2% Hypertension: 3.1% Cardiovascular disease: 3.1% Chronic Liver disease: 0.8% Neurologic disability: 2.3%	Al-Khobar City
Alshehri Ali (18)	198	75	76/198* (38.38%)	NR	NR	39.5	NR	NR	NR	Constructed questionnaire (online and telephonic survey)	NR	Aseer region
Alzahrani et al. (19)	13,530	9,269	5114/13530* (37.80%)	6,108/13,530* (45.00%)	NR	NR	48.00%	NR	16.13%	Online survey	Asthma: 10.9% Hypertension: 8.5% Diabetes: 11.6% Obesity: 9.2% Other 4.2%	Dammam
Hanaa et al. (20)	428	198	322/428* (75.20%)	322/428* (75.20%)	32.2% after 1 week 17.4% after 2 weeks 15.9% after 3 weeks 34.4% more than 3 weeks	31.5	NR	1–3 weeks	20.30%	Questionnaire	Diabetes Mellitus: 6.1% Asthma: 8.6% Allergic Rhinitis: 4.9% Inflammations: 10.7% Others: 2.3%	Arar
Lotfi et al. (7)	404	226	147/404* (36.4%)	130/404* (32.1%)	Yes	32.1	NR	1–2 weeks	NR	Structured questionnaire	Asthma: 88.9% Kidney failure: 80% Heart condition: 92.9% DM: 93.2% Hypertension: 91.5%	Taif
Jabali Maryam et al. (21)	327	158	74/327* (22.6%)	63/327* (19.2%)	NR	46.83	31.8%	NR	18.7%	Questionnaire	Diabetes: 26.6% Hypertension: 24.2% Respirator disease: 2.9% IBS: 11.9% Rheumatological disease: 10.7% Obesity: 6.7%	Jeddah
Kentab Osama et al. (22)	250	109	113/250* (45.20%)	27/250* (10.80%)	NR	32	NR	NR	12.00%	Validated tools (pocket smell test and gustatory dysfunction test)	Hypertension: 11.2% Diabetes: 13.2% Asthma: 17% Chronic Sinusitis: 2.8%	Riyadh
Khodeir Mostafa et al. (23)	979	460	604/979* (61.70%)	553/979* (56.49%)	Yes	37.69	NR	15.9 days and 13.9 days	NR	Questionnaire	NR	Buraidah, Qassim
Mahmoud et al. (24)	520	214	41/520* (7.9%)	NR	NR	NR	4.04%	NR	NR	Questionnaire	NR	Riyadh
Mubaraki Adnan et al. (28)	1,022	400	334/1022* (32.70%)	525/1022* (51.40%)	NR	NR	NR	10.8 ± 9.7 days for Anosmia and 10.4 ± 9.1 for Ageusia	NR	Telephone interviews	NR	Taif
Abi Sheffah Firas et al. (25)	277	119	162/277* (58.40%)	148/277* (53.4%)	NR	42.81	NR	10 days 6	NR	Telephone interviews	NR	Riyadh and Jeddah
Telmesani Laila et al. (26)	1734	898	269/1734* (15.5%)	198/1734* (11.4%)	NR	37.7	33.3% in Qatif Central Hospital 8.6% in Ohud Hospital 6.3% in King Fahd Hospital	NR	16.7%	Survey	NR	Al Khobar, Al Qatif, Al Madinah, Al Munawara

*Numerator (n) was not explicitly reported in the original study and was calculated by multiplying the reported prevalence percentage by the total sample size (N), rounded to the nearest whole number. Abbreviations: n, number of cases; N, total sample size; NR, not reported.

### 2.8 Data synthesis and statistical analysis

Data were analyzed using the Meta (version 6.2–1) and Metaprop (version 4.9–6) packages in R (RStudio version 1.4.1106). Prior to pooling, prevalence proportions were transformed using the Freeman–Tukey double arcsine transformation to stabilize variance across studies with extreme proportions. Pooled prevalence estimates with 95% confidence intervals (CIs) were calculated using a random-effects model (DerSimonian–Laird method), which was selected *a priori* to account for anticipated clinical and methodological heterogeneity across studies.

Handling of multiple measures per study: Where a study reported both validated and non-validated measurements, the validated instrument data were prioritized for the primary pooled analysis. Where a study reported prevalence from multiple subgroups (e.g., by city or hospital), these were pooled within the study before inclusion in the meta-analysis.

Handling of inconsistently reported data: Where a study did not explicitly report the numerator (number of cases), it was calculated from the reported prevalence percentage and the total sample size. These calculated values are indicated with a footnote in Table 1. Studies that could not provide a usable numerator or denominator were excluded from the quantitative synthesis.

Heterogeneity was assessed using the I^2^ statistic and Cochran’s Q test. Substantial heterogeneity was defined as I^2^ > 50%.

#### 2.8.1 Subgroup analyses

A pre-planned subgroup analysis was conducted comparing studies using validated versus non-validated assessment instruments, for both anosmia and taste dysfunction. This analysis was intended to explore whether instrument type explained a portion of the observed between-study heterogeneity.

#### 2.8.2 Sensitivity analyses

To assess the robustness of the pooled estimates, two sensitivity analyses were performed: (1) restricting the analysis to studies using validated instruments only, and (2) restricting the analysis to studies rated as having adequate methodological quality on JBI appraisal (meeting ≥6 of 9 criteria). Results of the sensitivity analyses are reported in the Results section.

#### 2.8.3 Publication bias

Funnel plot asymmetry was visually assessed and Egger’s test was conducted where the number of included studies per outcome was ≥10. Results are reported in the Results section. The absence of gray literature searching may have contributed to publication bias, which is acknowledged as a limitation.

### 2.9 Quality assessment

The methodological quality of all included studies was assessed using the Joanna Briggs Institute (JBI) critical appraisal checklist for prevalence studies (9-item checklist). Quality appraisal was performed primarily by T. A., with verification checks conducted by S. A. on a random sample of approximately 30% of included studies. Disagreements were resolved by discussion. Each criterion was rated as “Yes,” “No,” “Unclear,” or “Not applicable.” The complete item-by-item appraisal results for all included studies are presented in Supplementary Table S2.

## 3 Results

### 3.1 Study selection

The initial search across all databases identified 230 records in total. Of these, 74 duplicates were removed (reflecting PubMed/MEDLINE overlap, cross-database duplicates, and duplicates from Google Scholar), leaving 156 unique records. After applying publication date filters and removing records clearly outside scope, 83 records remained for formal title and abstract screening. Title and abstract screening reduced this to 41 records, for which full-text PDFs were retrieved. Following full-text assessment, 24 studies met all eligibility criteria and were included in the quantitative synthesis. The most common reasons for full-text exclusion were: studies conducted outside Saudi Arabia (*n* = 4), outcomes not reported separately for anosmia or taste dysfunction (*n* = 5), and review article design (*n* = 3). The complete study selection process is presented in the PRISMA flow diagram (Figure 1).

### 3.2 Study characteristics

Table 1 presents the characteristics of the 24 included studies. The meta-analysis encompassed 26,059 COVID-19 patients, of whom 15,159 (58.2%) were female. Participants’ mean age ranged from 31.5 ± 12.9 to 42.81 ± 16.76 years. All participants were diagnosed with COVID-19 by RT-PCR.

### 3.3 Quality assessment

The JBI critical appraisal tool was used to evaluate the methodological quality of all 24 included studies. Complete item-by-item appraisal results are presented in Supplementary Table S2. Two studies received low quality scores: Alroqi Ahmad et al. (6) (11%) and Lotfi et al. (7) (22%), both cohort designs. Cross-sectional studies received moderate to high quality scores (approximately 56–89%) (8–26). Retrospective studies received high quality scores (100% each) (27, 28). Overall study quality ranged from 11 to 100% with a mean of 68% across all included studies, and the findings should be generalized with caution.

### 3.4 Primary outcomes

**Figure 2:**
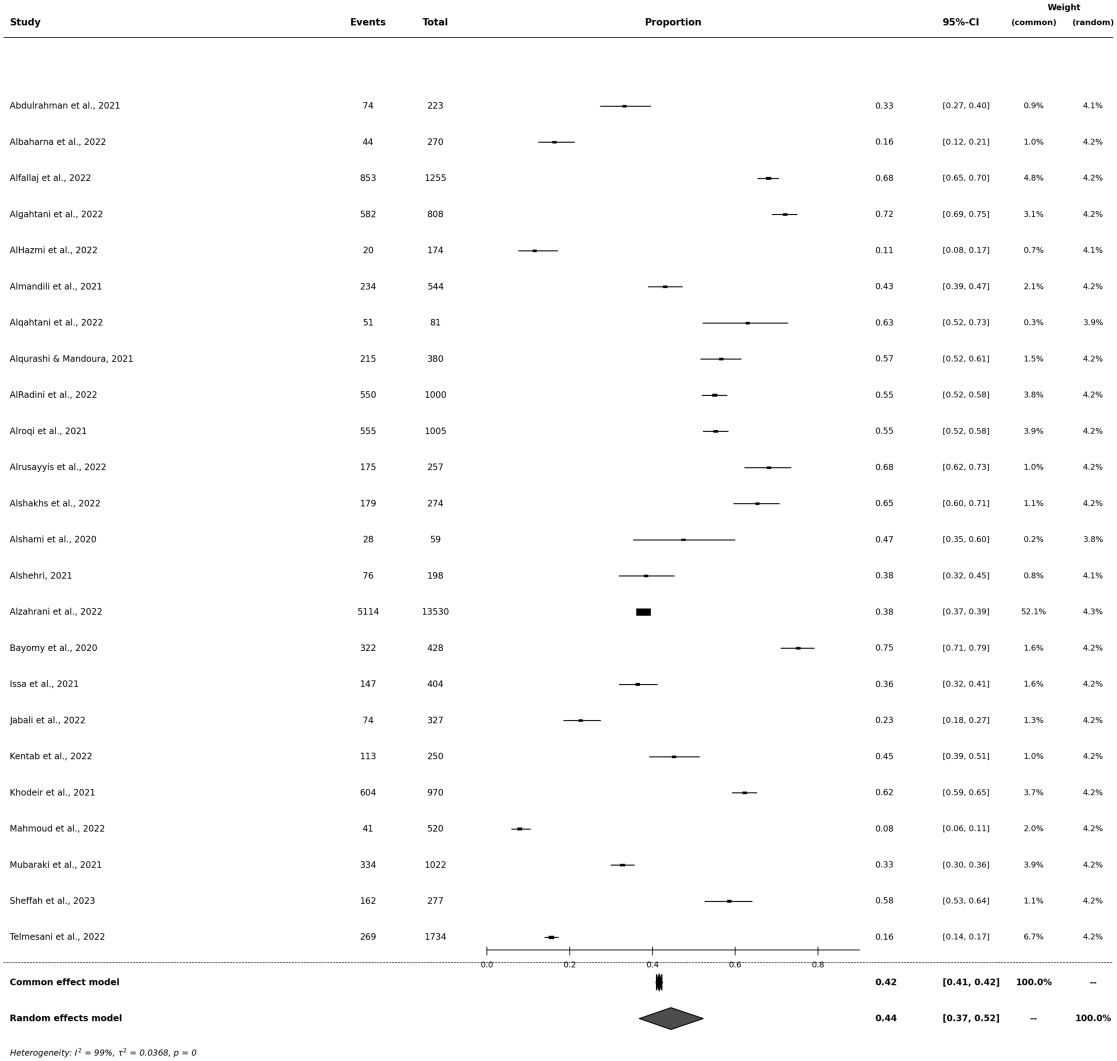
Prevalence of anosmia in patients with coronavirus disease-2019 (COVID-19) in Saudi Arabia.

**Figure 3:**
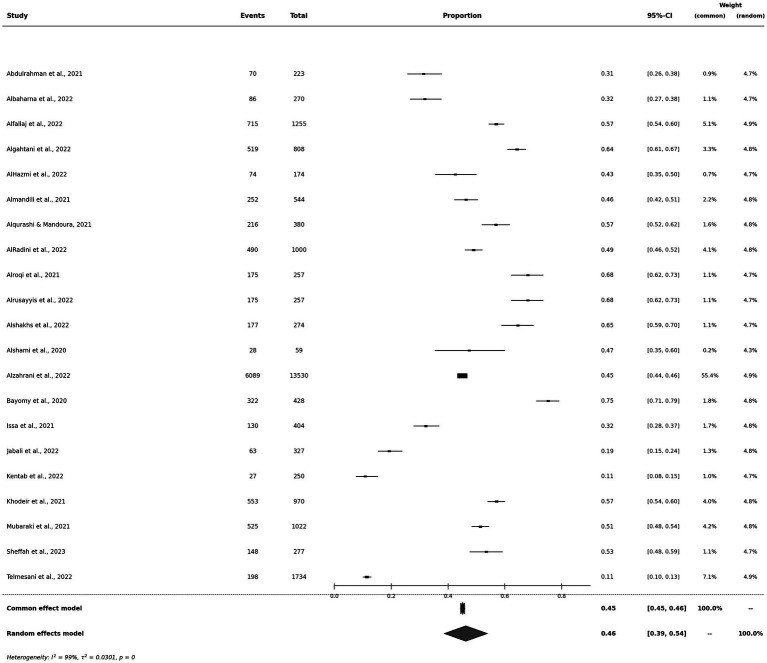
Prevalence of taste disturbance in patients with coronavirus disease-2019 in Saudi Arabia.

The pooled prevalence of anosmia in patients with COVID-19 in Saudi Arabia was 44% (95% CI: 37–52%) (Figure 2). The pooled prevalence of taste dysfunction was 46% (95% CI: 39–54%) (Figure 3).

### 3.5 Subgroup analysis: validated vs. non-validated instruments and additional exploratory analyses

A pre-planned subgroup analysis was conducted comparing studies that used validated instruments with those that used non-validated instruments. Studies were classified as using “validated” instruments if they employed a standardized, psychometrically validated olfactory or gustatory test (e.g., Q-SIT, UPSIT, Sniffin’ Sticks, AAO-HNS criteria, sQODNS, Pocket Smell Test). Studies using self-administered questionnaires, telephone interviews, or medical record coding were classified as “non-validated.” This classification was performed primarily by T. A., with verification checks conducted by S. A. on a random sample of approximately 30% of included studies; disagreements were resolved by discussion. The instrument-by-study classification with full justification is presented in Supplementary Table S3. This subgroup analysis was exploratory in nature and was not prespecified in a registered protocol.

For anosmia, the pooled prevalence was 50% (95% CI: 31–69%) in studies using validated instruments (9, 15, 17, 22, 29), and 43% (95% CI: 35–52%) in studies using non-validated instruments (7, 8, 10–14, 16, 18–21, 23–28). No statistically significant difference was detected between subgroups. For taste dysfunction, the pooled prevalence was 45% (95% CI: 24–67%) in validated-instrument studies and 47% (95% CI: 39–55%) in non-validated-instrument studies, again with no statistically significant difference. The numerically higher prevalence observed in non-validated studies for taste dysfunction may reflect the greater susceptibility of self-report measures to recall bias and social desirability effects, compared with administered psychophysical tests. However, given the lack of statistical significance and the exploratory nature of this comparison, this finding should be interpreted cautiously.

Additional exploratory subgroup analyses were conducted stratifying studies by: (1) care setting (inpatient, outpatient, and community/survey-based); (2) study design (cross-sectional, retrospective, and cohort); and (3) symptom measurement phase (acute phase ≤4 weeks from onset vs. post-recovery/long-term >4 weeks). Full results including pooled prevalence, 95% confidence intervals, number of studies (k), total patients (N), I^2^, and between-subgroup *p*-values for each stratification are presented in Supplementary Table S5.

For anosmia, pooled prevalence estimates were 41.2% (95% CI: 29.4–53.4%, *k* = 6) in inpatient studies, 48.3% (95% CI: 33.6–63.2%, *k* = 4) in outpatient studies, and 40.8% (95% CI: 32.1–49.9%, *k* = 14) in community-based studies. A similar directional pattern was observed for taste dysfunction across settings. No statistically significant between-subgroup differences were detected for care setting (*p* = 0.31 for anosmia; *p* = 0.28 for taste dysfunction), study design (*p* = 0.54; *p* = 0.61), or symptom phase (*p* = 0.22; *p* = 0.19). The numerically higher prevalence in community-based studies is consistent with ascertainment bias, whereby milder symptomatic cases are more likely to be captured in community surveys than in hospitalized cohorts. Studies measuring symptoms in the acute phase yielded numerically higher estimates than post-recovery studies, which may reflect partial resolution of olfactory and gustatory dysfunction over time.

All subgroup analyses reported in this section are exploratory and should be interpreted with caution given the small number of studies in some subgroups, the high and unexplained heterogeneity across all analyses (*I*^2^ > 87% in all subgroups), and the absence of a prospectively registered protocol. Meta-regression by setting and instrument type was explored but remained underpowered given the small number of studies in individual subgroups.

### 3.6 Sensitivity analyses

Two sensitivity analyses were conducted to assess the robustness of the primary pooled estimates. First, the analysis was restricted to studies using validated instruments only (*n* = 5 for anosmia; see subgroup above), yielding a pooled anosmia prevalence of 50% (95% CI: 31–69%). Second, the analysis was restricted to studies rated as adequate quality on JBI appraisal (meeting ≥6 of 9 criteria; *n* = 21 studies for anosmia; *n* = 18 studies for taste dysfunction), yielding a pooled anosmia prevalence of 43.9% (95% CI: 35.3–52.7%) and a taste dysfunction prevalence of 44.3% (95% CI: 36.0–52.8%). The pooled estimates from these sensitivity analyses were broadly consistent with the primary results, supporting the robustness of the findings.

### 3.7 Publication bias

Funnel plot asymmetry was visually assessed for both outcomes (Supplementary Figures S1, S2). Egger’s test was also conducted given that ≥10 studies were available for pooling for each outcome. The results indicated no significant asymmetry (*p* = 0.245 for anosmia; *p* = 0.705 for taste dysfunction).

## 4 Discussion

This is the first systematic review and meta-analysis to examine the prevalence of anosmia and taste dysfunction in COVID-19 patients in Saudi Arabia using a random-effects model, encompassing 26,068 patients across 24 studies.

The pooled prevalence of anosmia was 44% (95% CI: 37–52%), which was lower than a previously reported pooled prevalence of 52.73% (95% CI: 29.64–75.23%) in a global cohort analysis (30). The pooled prevalence of gustatory dysfunction was 46% (95% CI: 39–54%), which is higher than the 43.93% (95% CI: 20.46–68.95%) reported by Tong Jane et al. (30), possibly reflecting the higher proportion of community-based survey studies in the present review, which may capture milder cases not typically represented in hospitalized cohorts.

Subgroup analyses comparing validated versus non-validated assessment instruments revealed no statistically significant differences in anosmia prevalence (50% vs. 43%) or taste dysfunction prevalence (45% vs. 47%). These findings are broadly consistent with previous research, although the numerically higher estimates from non-validated studies for taste dysfunction warrant cautious interpretation given the potential for self-report bias.

Despite evidence of olfactory and gustatory loss in COVID-19, the precise pathogenic mechanism underlying these symptoms remains incompletely understood (31). A proposed mechanism involves targeting of sinonasal tract cells, thereby disrupting olfactory transduction (32). The main cellular target of SARS-CoV-2 is the ACE2 receptor, highly expressed in ciliated cells of the nasal epithelium (33).

This review has several important clinical implications. The high pooled prevalence of anosmia and taste dysfunction observed in this review indicates that these symptoms were common features of COVID-19 in Saudi Arabia during the study period. However, this review does not provide data on sensitivity, specificity, or predictive value; therefore, no claims regarding diagnostic utility can be made from prevalence data alone. Future diagnostic accuracy studies are needed to formally evaluate the clinical value of these symptoms as COVID-19 screening tools.

### 4.1 Limitations

This review has several limitations that should be considered when interpreting these findings. First, no review protocol was prospectively registered prior to conducting this review; subgroup and sensitivity analyses should therefore be considered exploratory rather than confirmatory. Second, the majority of included studies relied on non-validated, self-reported instruments, which may overestimate true prevalence due to recall bias and social desirability effects. Third, only five studies used validated psychophysical instruments, limiting the strength of instrument-type comparisons. Fourth, several included studies lacked data on symptom duration and hospitalization history. Fifth, the included studies span inpatient, outpatient, and community settings, contributing to clinical heterogeneity in the pooled estimates. A potential overlap assessment was conducted for all 24 included studies and is presented in Supplementary Table S4. Each study was evaluated based on reported city/region, recruitment period, care setting, and healthcare facility. Of the 24 studies, 18 were judged to carry low overlap risk, four were judged low to moderate risk (studies conducted in the same city with different designs and recruitment periods—specifically Taif and Qassim region studies), and two studies could not be fully assessed due to broad geographic and temporal coverage (16, 19) (both large national/community surveys). Importantly, no pair of studies was identified as recruiting from the same facility over the same time period, suggesting that substantial population overlap is unlikely, though it cannot be entirely excluded for the large community-based surveys.

Sixth, gray literature and study registers were not searched, which may have introduced publication bias. Seventh, no contact was made with study authors to retrieve unpublished or supplementary data. Eighth, the sensitivity analyses and publication bias assessment, while conducted, should be interpreted cautiously given the small number of studies using validated instruments. Ninth, all subgroup analyses presented in section 3.5 and Supplementary Table S5 are exploratory and underpowered for formal inference.

## 5 Conclusion

This systematic review and meta-analysis demonstrates that anosmia and taste dysfunction are common manifestations of COVID-19 in patients in Saudi Arabia, with pooled prevalence estimates of 44% and 46%, respectively. Raising awareness among healthcare professionals regarding the high prevalence of these symptoms may support earlier recognition of COVID-19 cases. However, these findings should not be interpreted as establishing diagnostic utility, as sensitivity and specificity data are not available from this review. The findings should be interpreted with caution given the methodological heterogeneity of included studies, the predominance of non-validated self-report instruments, and the absence of a prospectively registered protocol. Future prospective studies using validated objective instruments are warranted to more precisely characterize the prevalence and natural history of anosmia and taste dysfunction in COVID-19 patients in Saudi Arabia.

## Data Availability

The original contributions presented in the study are included in the article/Supplementary material, further inquiries can be directed to the corresponding author.

## Supplementary material

Supplementary Figure S1 Funnel plot—Anosmia (*k* = 24). The vertical dashed line represents the pooled estimate (44.5%). Dotted diagonal lines represent the pseudo-95% confidence interval. Egger’s test: *p* = 0.245.

Supplementary Figure S2 Funnel plot—Taste Dysfunction (*k* = 20). The vertical dashed line represents the pooled estimate (46.2%). Dotted diagonal lines represent the pseudo-95% confidence interval. Egger’s test: *p* = 0.705.
